# Diagnostic cytogenetic testing following positive noninvasive prenatal screening results of sex chromosome abnormalities: Report of five cases and systematic review of evidence

**DOI:** 10.1002/mgg3.1297

**Published:** 2020-05-08

**Authors:** Xiaolei Xie, Weihe Tan, Fuguang Li, Eric Carrano, Paola Ramirez, Autumn DiAdamo, Brittany Grommisch, Katherine Amato, Hongyan Chai, Jiadi Wen, Peining Li

**Affiliations:** ^1^ Department of Genetics Yale University School of Medicine New Haven CT USA; ^2^ Prenatal Diagnosis Center The Sixth Affiliated Hospital of Guangzhou Medical University Qingyuan People’s Hospital Qingyuan Guangdong China; ^3^ Diagnostic Genetics Sciences Program University of Connecticut Storrs CT USA

**Keywords:** chromosome microarray analysis (CMA), fluorescence in situ hybridization (FISH), karyotyping, mosaicism, noninvasive prenatal screening (NIPS), sex chromosome abnormalities (SCA)

## Abstract

**Background:**

Follow‐up cytogenetic analysis has been recommended for cases with positive noninvasive prenatal screening (NIPS) results. This study of five cases with numerical and structural sex chromosomal abnormalities (SCA) and a review of large case series of NIPS provided guidance to improve prenatal diagnosis for SCA.

**Methods:**

Following positive NIPS results for SCA, karyotype analysis, chromosomal microarray analysis (CMA), fluorescence in situ hybridization (FISH), and locus‐specific quantitative PCR were performed on cultured amniocytes, chorionic villi cells, and stimulated lymphocytes. Review of large case series was performed to evaluate the NIPS positive rate, follow‐up rate of cytogenetic analysis, positive predictive value (PPV) for major types of SCA, and relative frequencies of subtypes of major SCA.

**Results:**

Of the five cases with positive NIPS for SCA, case 1 showed a mosaic pattern of monosomy X and isodicentric Y; case 2 showed a mosaic pattern of monosomy X confined to the placenta; cases 3 and 4 had an isochromosome of Xq, and case 5 showed a derivative chromosome 14 from a Yq/14p translocation of maternal origin. Review of literature showed that mean positive rate of NIPS for SCA was 0.61%, follow‐up rate of cytogenetics analysis was 76%, and mean PPV for SCA was 48%. Mosaic patterns and structural rearrangements involving sex chromosomes were estimated in 3%–20% and 3% of SCA cases, respectively.

**Conclusion:**

These five cases further demonstrated the necessity to pursue follow‐up cytogenetic analysis to characterize mosaic patterns and structural abnormalities involving sex chromosomes and their value for prenatal genetic counseling. A workflow showing the performance of current NIPS and cytogenetic analysis for SCA was summarized. These results could facilitate an evidence‐based approach to guide prenatal diagnosis of SCA.

## Graphical abstract

we described five clinical cases with discordant or unexpected cytogenetic findings following positive NIPS results for sex chromosome abnormalities (SCA) and performed a systematic evidence review of large case series to evaluate the positive rate and positive predictive value of NIPS for SCA, the rate of follow up for cytogenetics analysis, and the relative frequencies of various subtypes of major SCA. This study further demonstrated the necessity and significance to pursue diagnostic cytogenetic analysis and provide evidence for genetic counseling and informative decision making to improve prenatal diagnosis of SCA.

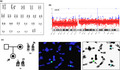

## 1 INTRODUCTION

Noninvasive prenatal screening (NIPS) for fetal aneuploidy has been performed using high‐throughput next‐generation sequencing on circulating cell‐free DNA in maternal plasma. NIPS has been highly accurate in predicting fetal trisomies 13, 18, and 21 but less accurate for sex chromosome abnormalities (SCA) (Liao et al., 2014; Norton et al., 2015; Xie et al., 2019). In an earlier study using positive predictive value (PPV) as a quality assurance measure, the PPV of NIPS based on follow‐up cytogenetic findings was 23% for monosomy X and 67% for XXY (Meck et al., 2015). The major problems in NIPS for SCA included high false‐positive rate (Kalafat, Seval, Turgay, & Koc, 2015; Zhang et al., 2019), discordant sex between screening results and ultrasound findings or postnatal phenotypes (Byers et al., 2019; Wang et al., 2015), variable comfort levels among prenatal genetic counselors in discussing various SCA conditions (Fleddermann et al., 2019) and the majority of patients declined invasive prenatal diagnosis upon posttest counseling (Ramdaney, Hoskovec, Harkenrider, Soto, & Murphy, 2018). A recent retrospective study of 10‐year data from a clinical cytogenetics laboratory noted underdetection of SCA in current prenatal practice (Chai et al., 2019). Better understanding of the causes of these problems could help to improve the efficacy of prenatal screening and follow‐up diagnosis for SCA.

Here, we described five cases with discordant or unexpected cytogenetic findings following positive NIPS results for SCA. We also performed a literature review to evaluate the positive rate of NIPS for SCA, follow‐up rate for cytogenetic analysis, the PPV of NIPS for major types of SCA, and relative frequencies of subtypes of major SCA from follow‐up cytogenetic analysis. These results further demonstrated the necessity of diagnostic cytogenetic analysis and their clinical value in prenatal genetic counseling and informative decision‐making.

## 2 METHODOLOGY

### 2.1 NIPS, Karyotyping, and FISH Analysis

NIPS for high‐risk pregnant women was ordered by obstetricians and performed by a commercial laboratory or in a hospital‐based clinical molecular laboratory as previously described (Xie et al., 2019). NIPS positive cases were offered genetic counseling with recommendation of follow‐up cytogenetic analysis. Karyotyping was performed on Giemsa–Trypsin–Wright's (GTW) banded metaphases for cultured amniocytes from amniocentesis, cultured fibroblast cells from chorionic villus sampling (CVS) or cultured lymphocytes from peripheral blood specimen following laboratory's standard protocols. FISH tests were performed using the AneuVysion tri‐color probes for the DXZ1 locus at centromere (cen) of X chromosome, the DYZ3 locus at Yq12 and the D18Z1 locus at 18cen on directly prepared cells (Abbott Inc. Des Plaines, IL).

### 2.2 Chromosome microarray analysis (CMA) and Quantitative PCR

Genomic DNA was extracted from cultured amniocytes, chorionic villus cells, and peripheral blood lymphocytes using the Gentra Puregene Kit (Qiagen, Valencia, CA). CMA was performed by array comparative genomic hybridization (aCGH) using Agilent SurePrint G3 Human CGH + SNP microarray (Agilent Technologies, Inc., Santa Clara, CA) as previously described (Li et al., 2011) or Affymetrix GeneChip System (ThermoFisher, Waltham, MA). The base designations were based on the February 2009 Assembly (GRCh37/hg19) of the UCSC Human Genome browser (http://genome.ucsc.edu/). Quantitative PCR to detect ***SRY*** gene and ***AZFa/b/c*** loci was performed on DNA extracted from cultured amniocytes using a quantitative PCR kit (Toujing, Shanghai, China).

### 2.3 Review of literature

Literature review was performed on large cases series with NIPS results and follow‐up cytogenetic findings for SCA. Thirteen relevant articles of NIPS case series were retrieved from PubMed using the following terms: “NIPS or NIPT and sex chromosome abnormality” (Chen et al., 2019; Garshasbi et al., 2019; Kornman et al., 2018; Liang et al., 2019; McLennan et al., 2016; Ramdaney et al., 2018; Suo et al., 2018; Xu et al., 2019, 2020; Xue et al., 2019; Zhang et al., 2017; Zheng et al., 2019; Zhou et al., 2019). To understand the outcomes from NIPS to cytogenetic analysis, the positive rate of NIPS for SCA, the follow‐up rate of NIPS positive cases selecting cytogenetic analysis postgenetic counseling, the PPV for major types of SCA, and the relative frequencies of subtypes for each major type of SCA were analyzed.

## 3 RESULTS

### 3.1 Cytogenetic and clinical findings from five cases

Five cases with positive NIPS results predicting SCA and discordant or unexpected follow‐up cytogenetic findings were selected; the laboratory results from NIPS to various cytogenetic analyses are summarized in Table 1.

**Table 1:** Follow‐up cytogenetic analysis on five cases with positive NIPS results predicting SCA

Cases	NIPS	Samples	Karyotype	FISH	CMA
1	Loss X	AF	mos 45,X[45]/46,X,idic(Y)(p11.3)[8]	nuc ish(DXZ1x1)[39]/(DXZ1x1,DYZ3x2)[19]/(DXZ1,DYZ3)x1[42]	arr[hg19](X)x1,(Y)x0 ~ 1
2	Loss X	CVS	46,XX	nuc ish(DXZ1x1)[62]/(DXZ1x2)[38]	arr[hg19](X)x1 ~ 2
3	Loss X	AF/PB	46,X,i(X)(q10)	*N*/A	arr[hg19] Xp22.33p11.21(481940–57931761)x1,Xq11.1q28(61931689–155235105)x3
4	Loss X	CVS	mos 46,X,i(X)(q10)[13]/45,X[7]	*N*/A	arr[hg19](X)x1[0.8]/(X)x1,Xq11.1q28(61781601–155208244)x2[0.2]
5	Presence Y	AF	46,XX,der(14)t(Y;14)(q12;p13)mat	ish der(14)t(Y;14)(DYZ1+)	arr[hg19]Yq12(59077673–59329950)x1

#### 3.1.1 Case 1

**Figure 1:**
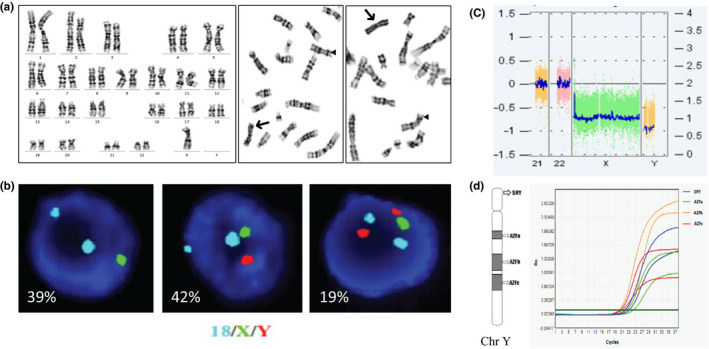
A mosaic pattern of monosomy X and isodicentric Y chromosome in Case 1. (a) Chromosome analysis of the culture amniocytes showed typical 45,X (left panel) and 46,X,idic(Y)(p11.3); a triangle points to normal X chromosome and an arrow points to idic(Y)(p11.3) (middle and right panels). (b) Result of FISH test using probes for chromosome 18 (aqua), X (green) and Y (red). The images from left to right showed monosomy X, XY and XYY, respectively. (c) The CMA result showed the loss of X chromosome and the presence of Y chromosome. (d) Result from a quantitative PCR test in duplicate showed positive result for the ***SRY*** gene and ***AZFa/b/c*** loci

A 27‐year‐old woman underwent NIPS at 15 weeks of gestation. The NIPS result showed z‐value of −6.88 for X chromosome which predicted the fetus had a complete or partial absence of one X chromosome. The pregnant woman selected amniocentesis for cytogenetic analysis. The karyotype from cultured amniocytes showed a mosaic pattern of monosomy X and isodicentric Y chromosome (Figure 1a). FISH test was performed using tri‐color probes for the DXZ1, DYZ3, and D18Z1 loci on directly prepared amniocytes. The analysis of 100 cells showed a mosaic pattern of monosomy X, XY, and XYY in 39%, 42%, and 19% of amniocytes, respectively (Figure 1b). The XYY pattern was explained by the isodicentric Y and the lack of XY pattern in cultured amniocytes was likely due to a low mitotic activity under in vitro cell culture. The CMA result showed the loss of X chromosome and the presence of Y chromosome (Figure 1c). The presence of Y chromosome was further confirmed by a quantitative PCR showing positive for the ***SRY*** gene and ***AZFa/b/c*** loci of the Y chromosome (Figure 1d). Follow‐up parental chromosome analysis showed normal karyotypes for the couple. Moreover, the ultrasound scan at 21 weeks of gestation showed a fetus with male genitalia. After genetic counseling, the couple decided to terminate the pregnancy by induced abortion at 27 weeks of gestation. Physical examination of the fetus showed external male genitalia without other apparent abnormalities. The couple refused further cytogenetic testing and pathology examination of the fetus.

#### 3.1.2 Case 2

A 36‐year‐old pregnant woman conceived by in vitro fertilization underwent NIPS at 14 weeks of gestation. The NIPS result indicated a high risk for monosomy X. Chromosome analysis was performed on cultured fibroblasts from CVS. Of the 20 metaphases analyzed and an additional 30 metaphases counted, no structural or numerical abnormality was noted and the karyotype was consistent with that of a normal female (46,XX). FISH test was performed using tri‐color probes for the DXZ1, DYZ3, and the D18Z1 loci on directly prepared chorionic villus cells. Of the 100 nuclei examined, 38% showed a normal female pattern of two signals each for the DXZ1 and D18Z1 probes and of no signal for the DYZ3 probe and 62% showed an abnormal pattern of monosomy X with one signal for the DXZ1 probe. This mosaic pattern of X/XX was confirmed by CMA on DNA extracted from directly dissected villi. After genetic counseling, the couple continued this pregnancy. Fetal polyhydramnios was detected by ultrasound during the third trimester and amniotic fluid reduction surgery was performed at 34 weeks of gestation. The baby was born vaginally at 40 weeks of gestation and physical examination was normal. Further karyotype and FISH testing on cultured lymphocytes from the baby's blood sample showed only 46,XX for a normal female pattern. These results indicated confined placental mosaicism for the 45,X/46,XX detected on villi cells.

#### 3.1.3 Case 3

A 33‐year‐old woman of first pregnancy underwent NIPS and the result showed an increased risk for loss of X chromosome. Prenatal chromosome analysis on cultured amniocytes and postnatal confirmatory chromosome analysis on cultured lymphocytes showed an abnormal pattern with an isochromosome of Xq: 46,X,i(X)(q10). The CMA on DNA extracted from blood leukocytes showed a 57.450 Mb deletion of Xp22.33‐p11.21 and 93.303 Mb duplication of Xq11.1‐q28. The baby was born via cesarean section at 40 weeks of gestation due to preeclampsia. At birth, the newborn baby was found to have difficulty feeding and lethargy; an echocardiogram test showed a normal result.

#### 3.1.4 Case 4

**Figure 2:**
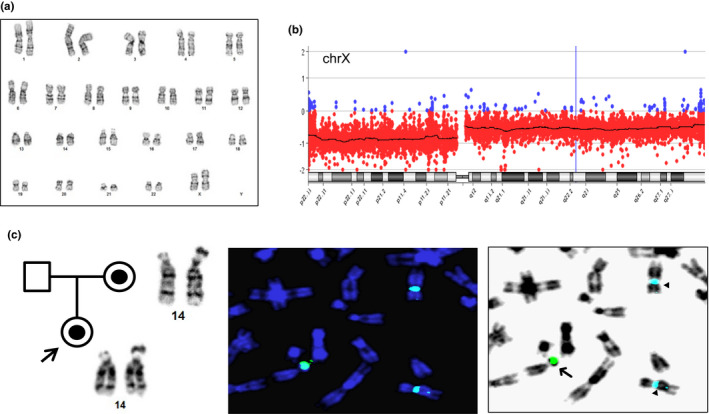
SCA detected in cases 4 and 5. (a) Chromosome analysis on CVS detected an isochromosome of Xq in case 4. (b) CMA result showed a deletion of Xp22.33‐p11.1 and Xq11.1‐q28 with different L2R in case 4. (c) The pedigree showed the transmission of a derivative chromosome 14 from mother to the fetus of case 5. FISH and reversed DAPI images showed DYZ1 signal on the short arm of a derivative chromosome 14 (arrow) and DXZ1 signal on two normal chromosome X (triangles)

A 24‐year‐old woman underwent NIPS at 11 weeks of gestation. The NIPS result showed an increased risk for monosomy X. Chromosome analysis was performed on cultured fibroblasts from CVS. Of the 20 metaphases examined, a mosaic pattern consisting of a loss of one X chromosome in seven cells and an isochromosome of Xq in 13 cells was noted (Figure 2a). CMA result showed an abnormal pattern with a 58.357 Mb deletion of Xp22.33‐p11.1 and a 93.427 Mb deletion of Xq11.1‐q28 with different log2 ratio (L2R) (Figure 2b); the L2R from these deletions indicated monosomy X in about 80% of cells and an isochromosome Xq in about 20% of cells. The fetal echocardiogram result at 20 weeks of gestation was normal. The baby was born vaginally at 38 weeks of gestation and physical examination was normal. The couple refused further cytogenetic testing on the baby.

#### 3.1.5 Case 5

A 35‐year‐old pregnant woman who underwent NIPS at 15 weeks of gestation indicated risk for ‘excess X signal and presence of Y signal’. An amniocentesis was performed at 17 weeks of gestation. Chromosome analysis on cultured amniocyotes showed a normal 46,XX female karyotype with an enlarged satellite on the short arm of a chromosome 14. FISH test was performed using dual color probes for the DXZ1 locus at Xcen and DYZ1 locus at Yq12. The examination of five metaphases noted the DYZ1 on the short arm of chromosome 14. This result indicated a derivative chromosome 14 from a translocation of Yq material onto the 14p (Figure 2c). CMA result revealed an XX female with an extra 252 Kb of Yq12 region containing the ***SPRY3*** and ***VAMP7*** genes. Due to the highly repetitive sequences in the distal Yq region, the exact size of the Yq material cannot be determined by CMA. Follow‐up parental chromosome and FISH studies showed a normal male karyotype in the father and a carrier of the derivative chromosome 14 in the mother, which indicated a maternal origin of this derivative chromosome 14. The ultrasound examination performed at 20 weeks of gestation showed that the fetus had normal female genitalia. The baby was born vaginally at 40 weeks of gestation and appeared to have normal external female genitalia.

### 3.2 Performance from NIPS to cytogenetic analysis

The review of literature found 13 studies of large case series with NIPS and cytogenetic findings. The mean positive rate of NIPS for SCA was 0.61% (ranged 0.43%‐0.79%), the mean follow‐up rate of NIPS positive for SCA after genetic counseling was 76% (ranged 65%‐86%), the PPV of NIPS for major types of SCA of 45,X, 47,XXX, 47,XXY, and 47,XYY were 31%, 61%, 73%, and 78%, respectively (Table 2). The subtypes of each major type of SCA and their relative frequencies were retrieved from four studies (Lau et al., 2014; Ramdaney et al., 2018; Suo et al., 2018; Xu et al., 2019) and summarized in Table 3. For typical monosomy X and variant subtypes, the relatively frequency was 67%, 20%, 10%, and 3% for 45,X, mosaic 45,X/46,XX, mosaic 45,X/46,XY and X chromosome rearrangement, respectively. For 47,XXX, 47,XXY, and 47,XYY, relatively frequencies for mosaic patterns were 7%, 3%, and 12%, respectively.

**Table 2:** The positive rate of NIPS for SCA, follow‐up rate and the PPV for major types of SCA

	Size(*n*)		SCA positive(*n*)	SCA detection rate (%)	Follow‐up Cytogenetics (*n*)	Rate of Follow‐ up (%)	Cytogenetic TP (*n*)	SCA PPV (%)	The PPV(%) of major SCA Types			
									45,X	47,XXX	47,XXY	47,XYY
McLennan et al. (2016)	5,267		58	1.10%	48	83%	18	38%	25%	33%	55%	75%
Zhang et al. (2017)	10,275		57	0.55%	33	58%	18	54%	29%	100%	78%	100%
Kornman et al (2017)		5,185	60	1.16%	49	82%	17	35%	20%	33%	50%	100%
Suo et al. (2018)	8,384		64	0.76%	64	100%	30	47%	32%	56%	60%	88%
Ramdaney et al. (2018)		NA	136	NA	64	47%	30	47%	29%	50%	69%	83%
Garshasbi et al. (2019)	11,223		29	0.26%	29	100%	25	86%	67%	67%	80%	100%
Liang et al. (2019)	94,085		390	0.41%	390	100%	182	47%	26%	62%	83%	75%
Zheng et al. (2019)	8,594		44	0.51%	33	75%	18	55%	44%	58%	100%	50%
Zhou et al. (2019)	17,894		95	0.53%	56	59%	24	43%	23%	63%	79%	33%
Xu et al. (2019)	32,931		140	0.43%	101	72%	57	55%	26%	85%	85%	69%
Xue et al. (2019)	57,204		295	0.52%	197	67%	79	40%	21%	64%	66%	77%
Chen et al. (2019)	42,910		147	0.34%	112	76%	37	33%	NA	NA	NA	NA
Xu et al. (2020)	31,515		225	0.71%	143	64%	61	43%	26%	65%	75%	83%
**Mean (95% CI)**	**—**		**—**	**0.61%** **(0.43%–0.79%)**	**—**	**76%** **(65%–86%)**	**—**	**48%** **(40%–56%)**	**31%** **(22%–39%)**	**61%** **(49%–73%)**	**73%** **(64%–82%)**	**78%** **(65%–91%)**

Note

Bold values indicates the mean and average value calculated from results of these large case series.

Abbreviations: PPV, positive predictive value; SCA, sex chromosome abnormalities; TP, true positive.

**Table 3:** The relative frequencies of subtypes of major SCA in prenatal diagnosis

	45,X				47,XXX		47,XXY		47,XYY	
	45,X	mos. X/ XX	mos. X/ XY	X Rearrang.	47, XXX	mos.	47,XXY	mos.	47,XYY	mos.
Lau et al. (2014)	1		2		4		2		1	
Suo et al. (2018)	9	1	1		5		6		6	1
Ramdaney et al. (2018)	10	1	1	1	3		11		5	
Xu et al. (2019)	6	6			15	2	17	1	11	2
**Total** **RF**	**26** **(67%)**	**8** **(20%)**	**4** **(10%)**	**1** **(3%)**	**27** **(93%)**	**2** **(7%)**	**36** **(97%)**	**1** **(3%)**	**23** **(88%)**	**3** **(12%)**

Abbreviations: mos, mosaicism; Rearrang, Rearrangement; RF, relative frequency.

## 4 DISCUSSION

Of the five cases presented in this study, cases 1–4 were predicted to be monosomy X by NIPS. Follow‐up cytogenomic analysis revealed a mosaic pattern with the presence of isodicentric Y in case 1, confined placental mosaicism of 45,X/46,XX in case 2, an isochromosome of Xq in case 3, and a mosaic pattern for monosomy X and an isochromosome of Xq in case 4. Mosaic 45,X/46,XX or 45,X/46,XY and isochromosome of Xq were considered variant types of Turner syndrome. Case 5 was predicated to have Y chromosome by NIPS; cytogenetic analysis detected a derivative chromosome 14 from a Yq/14p translocation. Since the mother carried this derivative chromosome, the fetus was expected as a carrier female. These five cases further demonstrated that various causes can contribute to the discordance between NIPS and cytogenetic results, including maternal mosaicism, confined placental mosaicism, DNA copy‐number variants, and chromosome translocations (Hartwig, Ambye, Sorensen, & Jorgensen, 2017; Zhang et al., 2019). Therefore, NIPS can only be used as a screening test not as a diagnostic test.

Studies have showed that the sensitivity and specificity of NIPS for SCA was lower than that for autosomal aneuploidies (Chai et al., 2019; Garshasbi et al., 2019; Suo et al., 2018). The overall positive rate of NIPS for SCA was 0.61%. The follow‐up rate for cytogenetic analysis on positive NIPS for SCA after genetic counseling was only 76%, which was lower than the 90%–100% follow‐up rate for trisomies 21, 13, and 18 (Liang et al., 2019; McLennan et al., 2016; Zheng et al., 2019). The low follow‐up rate may be due to relatively mild phenotypes of some SCA and the lack of confidence of genetic counselors in communicating SCA cases (Fleddermann et al., 2019; Wigby et al., 2016). A retrospective study noted that many patients refused further diagnostic tests following positive NIPS results for SCA as it was unlikely to affect pregnancy management (Ramdaney et al., 2018; Reiss, Discenza, Foster, Dobson, & Wilkins‐Haug, 2017). The overall PPV for fetal SCA by NIPS was 48%. When further categorized by major types of SCA, PPV was 31% for 45,X, 61% for 47,XXX, 73% for 47,XXY, and 78% for 47,XYY (Table 2). It is obvious that NIPS performed better in detecting sex chromosome gain than monosomy X.

The relative frequencies on subtypes of major SCA are summarized in Table 3. For a major type involving monosomy X, the relatively frequencies were 67% for classical type 45,X, 20% for mosaic X/XX, 10% for mosaic X/XY, and only 3% for X chromosome rearrangements. Current laboratory guideline recommended by American College of Medical Genetics for Turner syndrome estimated that the isochromosome of Xq accounted for 15%‐18% of Turner syndrome cases (Wolff, Van Dyke, & Powell, 2010), which indicated an underrepresentative of sex chromosome rearrangement in current prenatal diagnosis. For other major types 47,XXX, 47,XXY, and 47,XYY, the relatively frequency for mosaic pattern was in the range of 3%‐12%. Recent studies revealed that, for 45,X, mosaic 45,X/46,XX, and 47,XXX, the incidence among child‐bearing women were 1/47,305, 1/3,548, and 1/4,731, respectively, and the prevalence in adult population were 12/100,000, 76/100,000, and 45/100,000, respectively (Prakash et al., 2019; Samango‐Sprouse et al., 2016). These results indicated significantly more mosaic 45,X/46,XX and 47,XXX than typical Turner cases in adult population, which was consistent with underdetection of mosaic 45,X/46,XX and 47,XXX in prenatal diagnosis (Chai et al., 2019). The distinctive diagnosis of these typical and variant types of SCA could be achieved only by cell‐based cytogenetic analysis.

Different clinical manifestations of typical Tuner syndrome, mosaic 45,X/46,XX or 45,X/46,XY, and isochromosome Xq have been documented. Typical Turner syndrome patients present short stature and could experience aortic dissection and other congenital disorders predisposing to cardiovascular death from childhood to young adults (Al Alwan, Khadora, & Amir, 2014; Prakash et al., 2019). The presence of Y chromosome material in mosaic 45,X/XY has an increased risk of developing gonadoblastoma and virilization (Cools et al., 2011; Wu et al., 2017). Mosaic 45,X/46,XX cases detected in adulthood showed reduced penetrance with phenotypes of less short statue, normal reproductive lifespan and birth rate, and no reported cardiovascular complications (Jung et al., 2014; Tuke et al., 2019). Most isochromosome of Xq resulted from nonallelic homologous recombination between palindromic low copy repeats and LINE elements at a rearrangement hotspot in the proximal Xp region (Koumbaris et al., 2011). Patients with an isochromosome of Xq could have hypothyroidism and mild mental retardation and a significant increased risk of developing autoimmune thyroid disease and required thyroxine (Al Alwan et al., 2014; Elsheikh, Wass, & Conway, 2001). Characterization of different subtypes of Turner syndrome or other major SCA by cytogenetic analysis is important for genetic counseling and informative decision‐making on prenatal and postnatal management.

**Figure 3:**
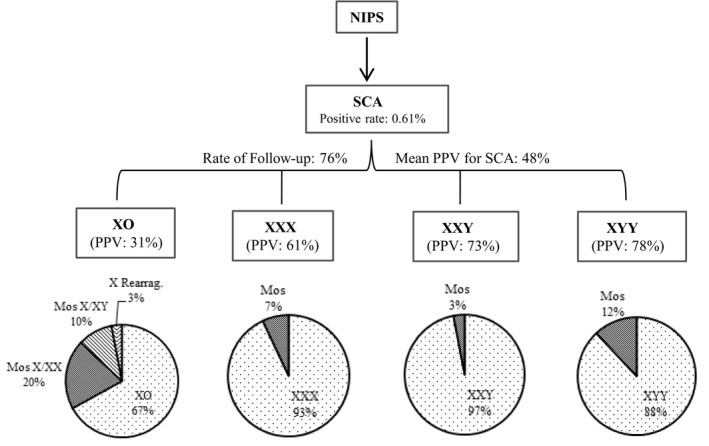
A flowchart showing the performance from NIPS to cytogenetic analysis for evidence‐based genetic counseling and informative decision‐making

A flowchart showing the performance from NIPS to cytogenetic analysis is presented in Figure 3. This information could be used in genetic counseling for informative and rational decision‐making by pregnant women. Follow‐up diagnostic cytogenetic analysis on positive NIPS for SCA should be recommended strongly to avoid underdetection and unclassified subtypes of SCA in prenatal diagnosis. It has been noted that complete follow‐up cytogenetic analysis was achieved by providing the test as gold standard (Suo et al., 2018), free of charge (Liang et al., 2019), and with insurance reimbursement of invasive procedure (Garshasbi et al., 2019).

## 5 CONCLUSION

We described five cases with discordant and unexpected cytogenetic findings following positive NIPS results for SCA and performed a literature review to show the positive rate of NIPS for SCA, follow‐up rate for cytogenetic analysis, PPV for major types of SCA, and the relative frequencies of subtypes of each major SCA. These results could facilitate an evidence‐based approach for better genetic counseling and informative decision‐making to improve prenatal diagnosis of SCA.

## CONFLICT OF INTEREST

The authors have no relevant disclosures or conflict of interest.

## AUTHORS’ CONTRIBUTIONS

X.X, W.T, F.L, E.C, P.R, A.D, G.B, K.A, and H.C. performed cytogenetic analysis on five cases. J.W and P.L. analyzed the laboratory data and reported results. X.X, E.C, P.R, and P.L. performed literature review. X.X, and L.P. wrote the manuscript. All authors reviewed and approved the manuscript.

## ETHICAL APPROVAL

All laboratory procedures were performed in a certified clinical laboratory. This project was categorized as a chart review retrospective study and deemed exempted from Institutional Review Board (IRB) approval and granted waiver of consent based on the policy of the Yale University IRB.

## Data Availability

The data that support the findings of this study are available on request from the corresponding author.
